# Stochastic dynamics of oxygen-hemoglobin binding: insight through facilitated exploration of an agent-based model

**DOI:** 10.3389/fphys.2026.1953414

**Published:** 2026-10-01

**Authors:** David M. Rubin, Andrea Kolkenbeck-Ruh, Xriz L. Richards, Penelope A. C. Keene, Svetlana M. King, Ann Z. George

**Affiliations:** 1 Biomedical Engineering Research Group, School of Electrical and Information Engineering, University of the Witwatersrand, Johannesburg, South Africa; 2 Cardiovascular Pathophysiology & Genomics Research Unit, Department of Physiology, School of Biomedical Sciences, Faculty of Health Sciences, University of the Witwatersrand, Johannesburg, South Africa; 3 Formerly at Department of Haematology and Molecular Medicine, School of Pathology, Faculty of Health Sciences, University of the Witwatersrand, Johannesburg, South Africa; 4 Prideaux Discipline of Clinical Education, Flinders Health and Medical Research Institute, College of Medicine and Public Health, Flinders University, Adelaide, SA, Australia; 5 Pathways to Fellowship, Royal Australasian College of Surgeons, Adelaide, SA, Australia; 6 Centre for Health Science Education, Phillip Tobias Building, Faculty of Health Sciences, University of the Witwatersrand, Johannesburg, South Africa; 7 Department of Education, University of Oxford, Oxford, United Kingdom

**Keywords:** agent-based model, dynamic equilibrium, oxy-hemoglobin, stochastic binding, threshold concept

## Abstract

**Background:**

Understanding oxygen–hemoglobin binding is fundamental to physiology and clinical practice. However, most students have an exclusively deterministic view of oxygen transport, including the misconception that hemoglobin simply loads oxygen in the lungs and unloads it at the tissues. Developing a conceptual understanding requires appreciation of stochastic molecular interactions, dynamic equilibrium, and population-level averaging of microstates, concepts that are abstract, difficult to grasp, and not typically taught in medical physiology courses.

**Methods:**

In this mixed methods study, we developed an agent-based model (ABM) in NetLogo to visualize oxygen–hemoglobin binding as a stochastic process. Based on a published quantitative model describing the distribution of hemoglobin bound states as a function of *P*O_2_, the ABM allows users to explore the effect of changing *P*O_2_ and hemoglobin number while observing stochastic changes in molecular states. Thirty-one second-year medical students participated in a facilitated computer laboratory session and completed pre- and post-intervention quizzes comprising 18 true/false questions. Quantitative data were analyzed using the McNemar test and a paired-samples t-test. Eight days after the facilitated session and quiz completion, four focus groups comprising 28 participants were conducted, and the focus group transcripts underwent inductive and deductive thematic analysis to identify common themes.

**Results:**

The ABM reproduced the expected oxyhemoglobin equilibrium curve and demonstrated stochastic fluctuations that diminished with increasing numbers of hemoglobin molecules. Quantitative analysis showed that performance improved on all 18 quiz items, with 11 showing statistically significant gains. Mean scores increased from 54.1% to 83.2% (mean difference 29.0%; t(30)=8.7, p=10^-9^; d_z_=1.56). Qualitative analysis identified three themes: knowledge construction through active exploration, affective responses associated with intellectual engagement, and mechanistic understanding of oxygen-hemoglobin binding. Findings demonstrated that participants replaced static mental models with an understanding of stochastic oxygen binding and recognized oxygen saturation as a population average rather than the behavior of individual hemoglobin molecules.

**Conclusion:**

Facilitated agent-based modeling substantially improved conceptual understanding of oxygen–hemoglobin binding. By interactively exploring stochastic molecular dynamics, the intervention promoted active learning, corrected persistent misconceptions, and may provide a transferable framework for understanding emergent physiological phenomena.

## 1 Introduction

Assessment of a patient’s oxygenation status is fundamental to clinical management, making oxygen transport a core topic in medical physiology. Routine measurement of arterial oxygen saturation by pulse oximetry has transformed clinical practice by providing continuous, non-invasive monitoring, leading to its description as the fifth vital sign (Kamat, 2002; Neff, 1988; Tozzetti et al., 2009). Although arterial blood gases (ABGs) remain the reference standard because they provide additional measurements such as *P_a_*O_2_, pulse oximetry has become an important tool for monitoring oxygenation in most clinical settings.

Understanding oxygen transport requires a shift from a static, deterministic view of oxygen carriage to one based on stochastic, dynamic molecular interactions. Oxygen molecules are in continuous random thermal motion, binding to and dissociating from hemoglobin at rates determined by molecular concentrations until a dynamic equilibrium is established (Perutz, 1978).

The relationship between oxygen saturation, *S*_O2_, and partial pressure of oxygen, *P*O_2_, is generally termed the oxyhemoglobin dissociation curve, or more rarely (Chou and Lee, 2023), the oxyhemoglobin association curve. For reasons that will become evident in the context of stochastic processes described in this paper, we will use oxyhemoglobin equilibrium curve, a term used frequently in earlier papers (Pauling, 1935; Perutz, 1978; O’Riordan, 1986; Vandegriff et al., 1998). Its characteristic sigmoidal shape reflects cooperative oxygen binding and modulation by factors such as pH, temperature, Cl^–^, 2,3–BPG, and CO_2_ (Bohr et al., 1904; Perutz, 1978).

Notwithstanding the pioneering studies of oxyhemoglobin by Hüfner (1890); Hill (1910); Adair et al. (1925) and others, the molecular basis of cooperativity, including the nature of the transitions between relaxed (R) and tense (T) states that determine binding affinities, is ongoing (Ackers, 1980; Johnson et al., 1992; Vorger, 1999; Klotz, 2002; Eaton et al., 2007; Farsaci et al., 2019; Lavrinenko et al., 2021, Lavrinenko et al., 2023; Chou and Lee, 2023).

Our experience suggests that many physiology students develop an unrealistically static mental model of oxygen transport in which hemoglobin molecules are “loaded” with oxygen in the lungs and simply transport it to the tissues for unloading. We refer to this as the “goods train” misconception. Students also frequently fail to distinguish between PaO_2_, which reflects dissolved oxygen, and S_O2_, which represents the fraction of hemoglobin binding sites occupied by oxygen. As a result, many resort to rote learning of the oxyhemoglobin equilibrium curve without understanding the underlying processes. Similar misconceptions have been reported by other investigators (Breckler and Yu, 2011; Michael et al., 1999; Cliff, 2006a, Cliff, 2006b).

These misconceptions are consistent with broader challenges in physiology education. Physiology is widely regarded as conceptually difficult because of both its intrinsic complexity and the way it is commonly taught (Michael, 2007). Misconceptions are particularly prevalent in respiratory physiology (Michael et al., 1999; Badenhorst et al., 2016), and conceptual understanding often fails to persist or transfer to clinical reasoning (Wilhelmsson et al., 2013).

Joyner et al. (2011) suggest that physiology educators should prioritize the understanding of physiological processes over polished visual presentations and the memorization of facts, which can obscure the uncertainties and gaps in our knowledge. They suggest that students should be offered robust intellectual engagement that facilitates acknowledgment and exploration of these uncertainties.

A range of educational approaches have been proposed to improve understanding of oxygen transport, including mathematical models (Khee-Shing Leow, 2007), kinesthetic activities (Breckler and Yu, 2011; Milanick, 2021), case-based learning (Cliff, 2006b, Cliff, 2006a), and a variety of analogies (Gomez-Cambronero, 2001). However, these approaches do not explicitly emphasize the stochastic dynamics that underpin oxygen-hemoglobin binding.

In this paper, we describe an active learning approach that introduces students to the stochastic thermodynamics of oxygen-hemoglobin binding. Our aim is to replace static mental models with a dynamic understanding of oxygen transport, thereby improving interpretation of pulse oximetry and ABG measurements while providing a conceptual framework that may be transferable to other areas of physiology and biochemistry.

## 2 Methods

We developed an agent-based model (ABM) (Wilensky, 1999a; Blikstein et al., 2005) to enable students to explore the stochastic nature of oxygen binding to hemoglobin (Hb). ABMs are decentralized computational models in which the individual entities (agents) engaging in the behavior are given behavioral attributes, which are frequently probabilistic and depend on factors such as their current state, their interactions with other agents, and their positions in the environment. The behavior of individual agents are typically governed by simple rules and may sometimes be unpredictable, but when aggregated, result in emergent, and frequently complex and surprising behavior. Examples include ant colonies, flocks of birds and the behavior of road traffic, where each ant, bird or vehicle is an agent, and the overall behavior emerges from their interactions (Resnick, 1997).

Our medical school offers a hybrid curriculum design – a six-year medical program with most students entering directly from high school, and a four-year graduate entry curriculum for students who have a suitable bachelor’s degree as a minimum entry requirement. In this study, participants were second-year medical students in the six-year program who were currently engaged in their full-year human physiology course at the University of the Witwatersrand, Johannesburg. They engaged with the model during a facilitated intervention led by the principal investigator. The study employed a mixed-methods design comprising quantitative analysis of pre- and post-intervention concept quizzes, followed by qualitative thematic analysis of focus group discussions in terms of institutional ethics approval.

Researcher reflexivity: The multidisciplinary research team included academics in Biomedical Engineering, Physiology, Medical Education, Hematology, and an educational researcher from Flinders University who contributed an external perspective to the qualitative analysis. The team’s disciplinary backgrounds and the lecturer–student relationship were recognized as potential influences on data collection and interpretation and were considered throughout the analysis through ongoing discussion and reflection. As the principal investigator and intervention facilitator was not one of the physiology lecturers, the potential for an adverse power dynamic was reduced.

### 2.1 Development of an agent-based model

In order to develop an ABM that facilitates exploration of the stochastic behavior of oxygen and hemoglobin interaction, it is necessary to have a mathematical model that produces realistic fractions of the five *n*-bound states of hemoglobin as a function of *P*O_2_, such that their aggregated behavior is consistent with the oxyhemoglobin equilibrium curve.

The theory of Chou and Lee (2023), after Adair et al. (1925), represents such a model which compares favorably with experimental data (Gill et al., 1987). The model comprises a set of formulae, with accompanying coefficients that have been calibrated to a specific oxyhemoglobin equilibrium curve with a *P*_50_ = 27 mmHg. This allows for the determination of the fractions of the bound states at any given $PO_{2}$ value. When these fractions are treated as probabilities, which is consistent with the stochastic behavior of this process, it becomes possible to develop a suitable ABM.

We have adopted the Chou and Lee (2023) model as the basis for our ABM. In order to achieve a more compact mathematical representation, we have condensed all five of their equations for the fractional populations of bound states implicit in the Adair et al. (1925) formulation, $f_{n}(PO_{2})$, as a function of $PO_{2}$ for each integer value of *n* = 0 to 4, into a single equation as follows:

(1) $$f_{n}(PO_{2})=\frac{A_{n}{\left(PO_{2}\right)}^{n}}{A_{0}+A_{1}(PO_{2})+A_{2}{\left(PO_{2}\right)}^{2}+A_{3}{\left(PO_{2}\right)}^{3}+A_{4}{\left(PO_{2}\right)}^{4}}$$

or expressed more compactly,

(2) $$f_{n}\left(PO_{2}\right)=\frac{A_{n}{\left(PO_{2}\right)}^{n}}{\sum_{i=0}^{4}A_{i}{\left(PO_{2}\right)}^{i}}$$

where (*P*O_2_)^0^ ≡ 1 at *P*O_2_ = 0, and where the coefficients, *A_n_* for *n* = 1 to 4, as determined by Chou and Lee (2023) (which they refer to as a, b, c, and d), together with the introduction of *A*_0_ = 1 for *n* = 0, appear in **Table 1**.

**Table 1:** Values of *A_n_* for *n* = 1 to 4 (data sourced) from Chou and Lee (2023), and *A*_0_, where *n* is the number of oxygen molecules bound to hemoglobin.

*n*	*A_n_*
0	1
1	0.0183177175
2	0.0008807801
3	0.0000028190
4	0.0000022948

We developed the ABM in NetLogo 6.4.0 (Wilensky, 1999b). We introduced a set of sliders in the model that allows the user to set both a *P*O_2_ value from 0 to 300 mmHg, and the number of hemoglobin molecules in the simulation ranging from 0 to 1000.

At every time step, each Hb molecule takes on one of the bound states with a probability determined by the fractional value at the prevailing *P*O_2_ calculated from Equation 1 or Equation 2, using the coefficients, *A_n_*, shown in **Table 1**. The bound states are represented by assigned colors: 0-bound (purple), 1-bound (blue), 2-bound (yellow), 3-bound (orange) and 4-bound (red).

During a simulation run, the Hb molecules change color on every time tick based on their probabilistically determined bound state. The probabilistic nature of the states ensures that no simulation run will be identical to any other. The Hb molecules also undergo small, random translational movements to provide a more realistic impression of molecular dynamics by mimicking Brownian motion.

The % Hb saturation, *S*_O2_, is calculated from the ratio of total number of oxygen bound sites on Hb to the total number of oxygen binding sites on Hb expressed in Equation 3 as follows:

(3) $$S_{O2}=\frac{Hb_{1}+2Hb_{2}+3Hb_{3}+4Hb_{4}}{4\sum_{n=0}^{4}Hb_{n}}\times 100$$

where *Hb_n_* represents the number of Hb molecules in the n-bound state.

It is important to note that our NetLogo ABM determines the saturation from actual counts of Hb molecules in various bound states which update stochastically with every time tick, rather than directly from the deterministic fractions calculated from the Chou and Lee (2023) formulae. This ensures that students can appreciate the stochastic nature of the process by observing the rapidly varying value of *S*_O2_ and the dynamically varying histogram of states.

An additional feature allows the operator to toggle a switch to render the oxygen molecules visible. This feature is purely to remind users of the oxygen molecules in solution – the oxygen molecules do not interact with Hb molecules in the program, and the actual numbers of oxygen molecules relative the number of Hb molecules is not physiologically based. The oxygen molecules appear as small green filled circles which mimic random thermal motion. Moving the *P*O_2_ slider changes the number of oxygen molecules, however the actual number is purely for representational purposes. Moreover, the model does not facilitate simulations of the actual interactions between Hb and the oxygen ligands. A simplified block diagram of the ABM’s program flow is shown in **Figure 1**.

**Figure 1:**
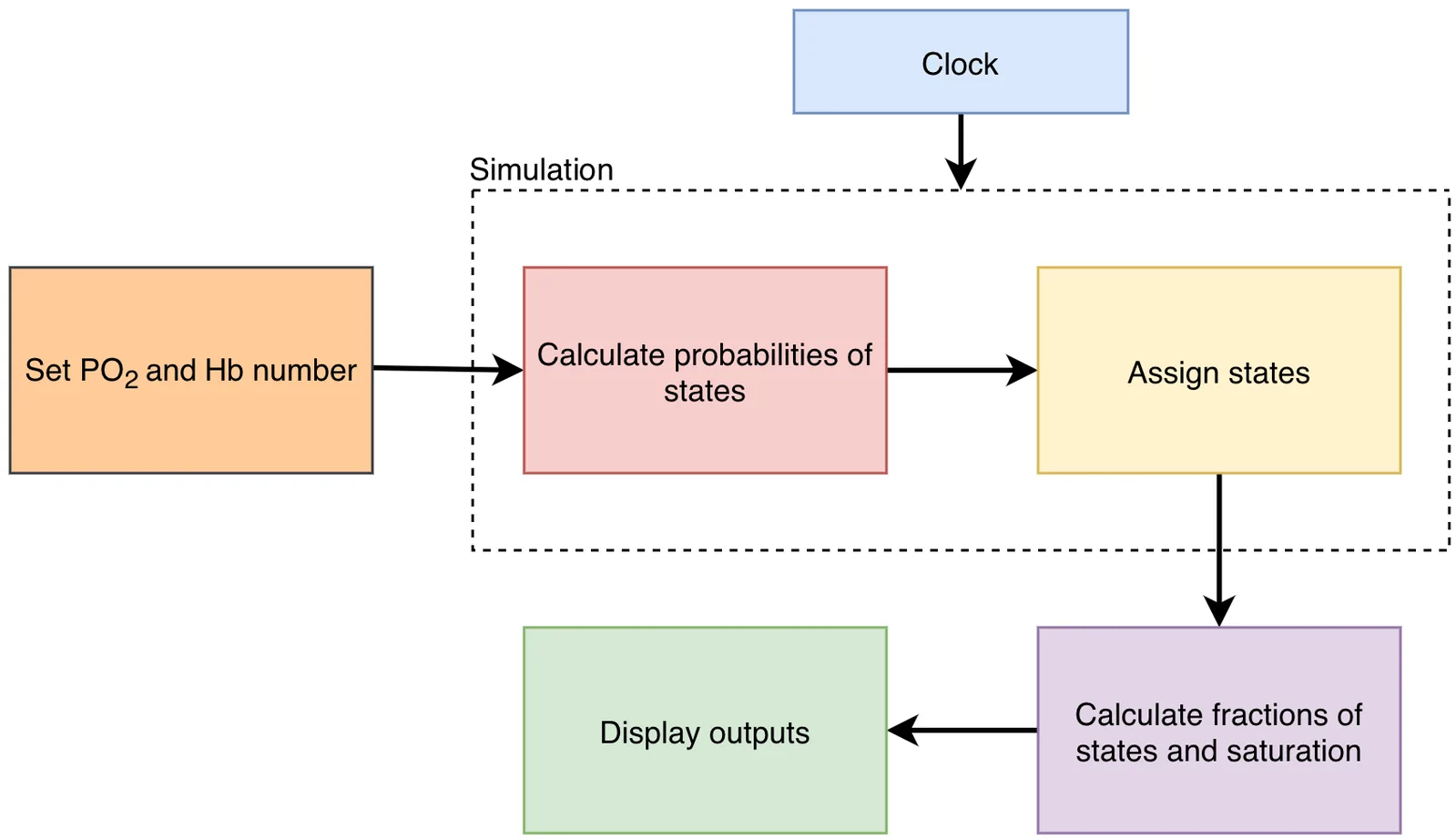
Simplified block diagram showing the operation of the ABM. The number of Hb molecules and *P*O_2_ are set. From the *P*O_2_ value, probabilities are calculated and states assigned on every clock tick. The fractions of states and oxygen saturation are calculated and displayed in various formats.

The ABM dashboard is shown in **Figure 2**. The large panel on the right shows the color-coded Hb molecules which are continuously updating their state with each time step. The *P*O_2_ and Hb number sliders are placed at the top left. Immediately below this, there is a display panel showing the percentage of Hb molecules in each oxygenation state as color-coded time series. Below this, on the left, the overall oxygen saturation is shown as a digital display and a graphical time series. Immediately to the right of this, a histogram is displayed showing each of the five states. The oxygen toggle, setup button, and Go/Stop button, are placed at the bottom left.

**Figure 2:**
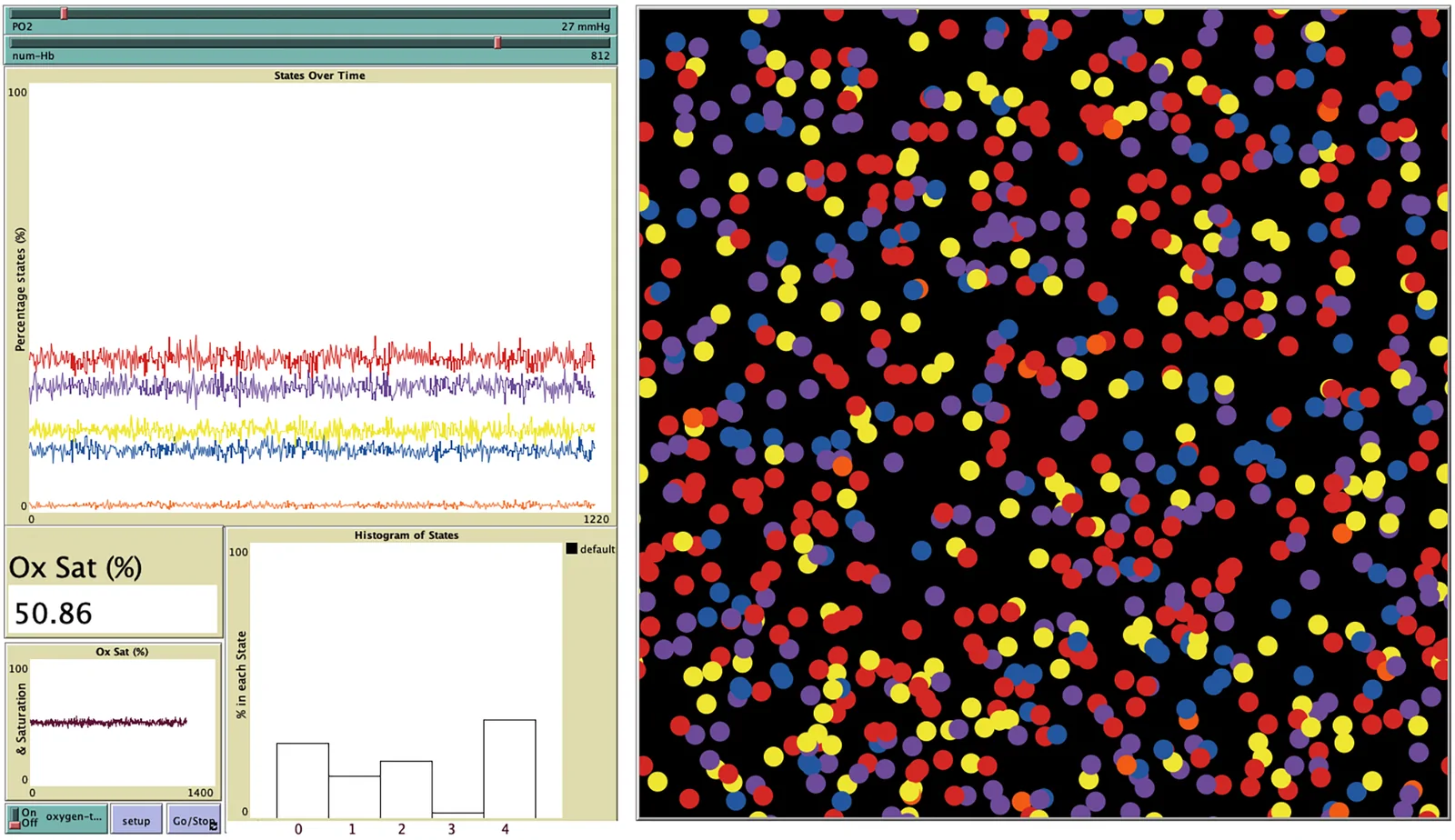
ABM dashboard in NetLogo showing the agent-based model operating at *P*O_2_ = 27 mmHg. *P*O_2_ and Hb sliders are at the top left. Below these, a graphical display shows the color-coded fractions of the five hemoglobin (Hb) states over time. Overall oxygen saturation is shown numerically and graphically (Ox Sat (%)), as well as a dynamic histogram of the five Hb states. The main display on the right shows Hb molecules (agents) changing between the five color-coded oxygenation states.

### 2.2 The facilitated computer laboratory session and quizzes

Thirty one volunteer participants from the medical physiology class of about 250 students convened for the facilitated laboratory session which took place in one of the computer laboratories at the medical school over a duration of about 80 minutes. All participants were second-year medical students on the six-year program at the University of the Witwatersrand, Johannesburg.

The students who participated in this study had recently completed the respiratory component of the second-year physiology curriculum. Due to the extensive number of respiratory physiology learning objectives prescribed in the course, the oxyhemoglobin dissociation curve (the term used in the course) was covered in a single 45-minute lecture. During this lecture, students were introduced to the four oxygen binding sites on hemoglobin, with a discussion on how the binding of oxygen molecules increases hemoglobin’s affinity for subsequent oxygen binding in a process known as cooperativity. However, the concepts of dynamic equilibrium, stochasticity and microstates were not examined in this lecture. The instruction included the fundamental physiological principles underlying oxygen transport. Specifically, students were required to:

- briefly describe the chemical nature of hemoglobin responsible for oxygen binding.
- compare the proportion of oxygen dissolved in plasma with that bound to hemoglobin.
- explain how dissolved oxygen and hemoglobin-bound oxygen are influenced by factors such as arterial oxygen partial pressure, hemoglobin concentration, oxygen-binding capacity, and oxygen saturation.
- describe the oxygen dissociation curve with reference to the oxygen-carrying capacity of hemoglobin; the effects of temperature, pH and carbon dioxide on oxygen binding (the Bohr effect); and the role of 2,3-diphosphoglycerate (2,3-DPG).

Each volunteer was provided with a participant information leaflet. After sufficient time to read the leaflet covering both aspects of the study, namely the facilitated activity with quizzes, and the focus groups, the students were offered an informed consent document for their signature in terms of our institutional review board ethics approval: HREC(Medical) protocol number M250831. Each participant was given a modest lunch voucher for an on-campus food vendor as required in terms of our ethics committee’s policy of compensation for time, inconvenience and expense.

Participants were asked to draw a number out of a “hat” which only they would know and they would write it on both the pre and post quiz. They were then asked to complete the 18 true/false questions of the pre-quiz. The questions tested understanding of the stochastic process and encompassed stochastic behavior, dynamic equilibrium, and averaging effects. Participants were carefully instructed not to write any identifying information on the quizzes. The questions are available in the **Supplementary Material**.

Each participant had their own workstation with NetLogo 6.4.0 and the oxyhemoglobin ABM installed. The facilitator, who was the principal investigator, displayed a running version of the software on a large digital projection screen.

Participants were encouraged to experiment with the *P*O_2_ and Hb sliders to investigate the behavior of the ABM. The environment was informal and participants and facilitator engaged in discussion and explanations. Participants were asked to estimate saturation values for a range of *P*O_2_ settings and these were plotted by the facilitator on a display chart. Most of the interaction related to the unexpected (from the participants’ perspective) behavior of oxygen-hemoglobin binding. At the end of the session, participants were again asked to complete the questionnaire after writing their unique and private number key on the paper. This facilitated correlating the pre and post-quizzes while maintaining anonymity.

### 2.3 Focus groups

Twenty eight of the original 31 participants reconvened for focus group sessions, which were conducted eight days after the facilitated session, to allow for reflection. The participants were divided into four focus groups, each comprising 6 to 8 participants. Participants were asked to choose an arbitrary alias for the purpose of anonymity on the transcript and each student wrote their alias on a sticker which they wore for ease of reference during the session.

The focus groups were led by four of the authors, DR, AK-R, AG and XR, only one of whom, AK-R, was the physiology lecturer, thus largely mitigating the potential for an adverse power dynamic affecting the results. The sessions ran for about one hour, and were audio-recorded using both Microsoft Teams on a laptop and a separate digital recorder. The Teams recording was automatically transcribed and the principal investigator listened to both recordings for each group and made corrections to the auto-transcription as required. Each focus group leader then listened to the recordings again and made further corrections to the transcript until they were satisfied that it represented a faithful reproduction of the session.

The focus group discussions were guided by a small set of semi-structured questions:

- How confident did you feel about the O_2_-hemoglobin equilibrium curve before the intervention?”
- How did the simulation exercise help you to understand the concepts?
- Are there aspects of the topic that still feel unclear?
- Did improved understanding happen gradually or was it a sudden “light-bulb” moment?
- Do you think you can apply what you have learned in other physiological contexts?
- How would you explain these concepts to a fellow student who finds it difficult?

The transcripts were then coded and themes developed using MAXQDA PLUS (release 26.0.0, VERBI, Berlin) by DR according to the general scheme of Clarke and Braun (2013). We primarily follow an inductive approach, with a deductive element for the specific aspects of the stochastic behavior. As these were performed separately, this constitutes a hybrid rather than an abductive approach.

We began by inductively identifying themes that emerged through iterative reading, analysis and coding of the focus group transcripts. This was initially performed on all four focus groups by the principal investigator. These were then reviewed by two of the other authors, AG and SK, who have extensive experience in qualitative research. Participants were further anonymized in the analysis by replacing their alias with an arbitrarily assigned number within focus groups designated A, B, C and D.

Whereas the inductive themes capture broad patterns in the participants’ experiences and offer wide insights into the educational impact of this approach to teaching and learning in physiology, the deductive components were specific to the scientific basis of this study, namely the stochastic processes underlying oxygen carriage by hemoglobin.

## 3 Results

### 3.1 Model verification

We examined the performance of the ABM to establish if it was “fit for purpose” as an active learning platform for stochastic processes in oxyhemoglobin binding. We began by examining the displays which showed Hb molecules updating on every time tick, while the histogram of the five states showed variability which increased with fewer Hb molecules. The color-coded time series representing the fraction of each state showed appropriate variability which increased with reduction of Hb molecules, as did the overall saturation as shown in **Figure 3**. The changes in distribution of states with varying *P*O_2_ is evident in **Figure 4**.

**Figure 3:**
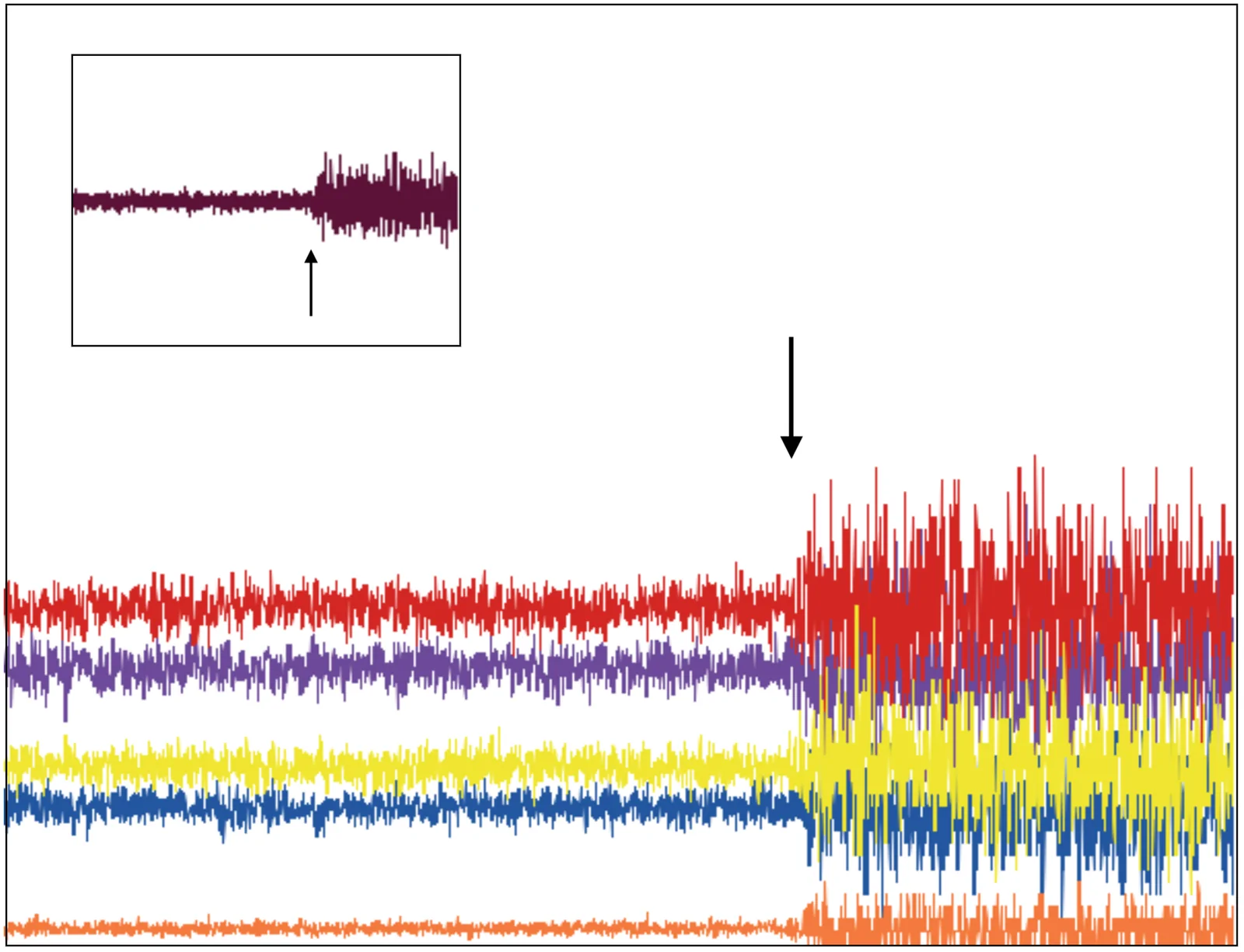
Time series of each of the five Hb bound states. The simulation starts with 1000 Hb molecules and part way through the run (arrow), the number of Hb molecules is reduced to 75, resulting in greater variance but with the same average. The inset shows the same effect on the percentage saturation.

**Figure 4:**
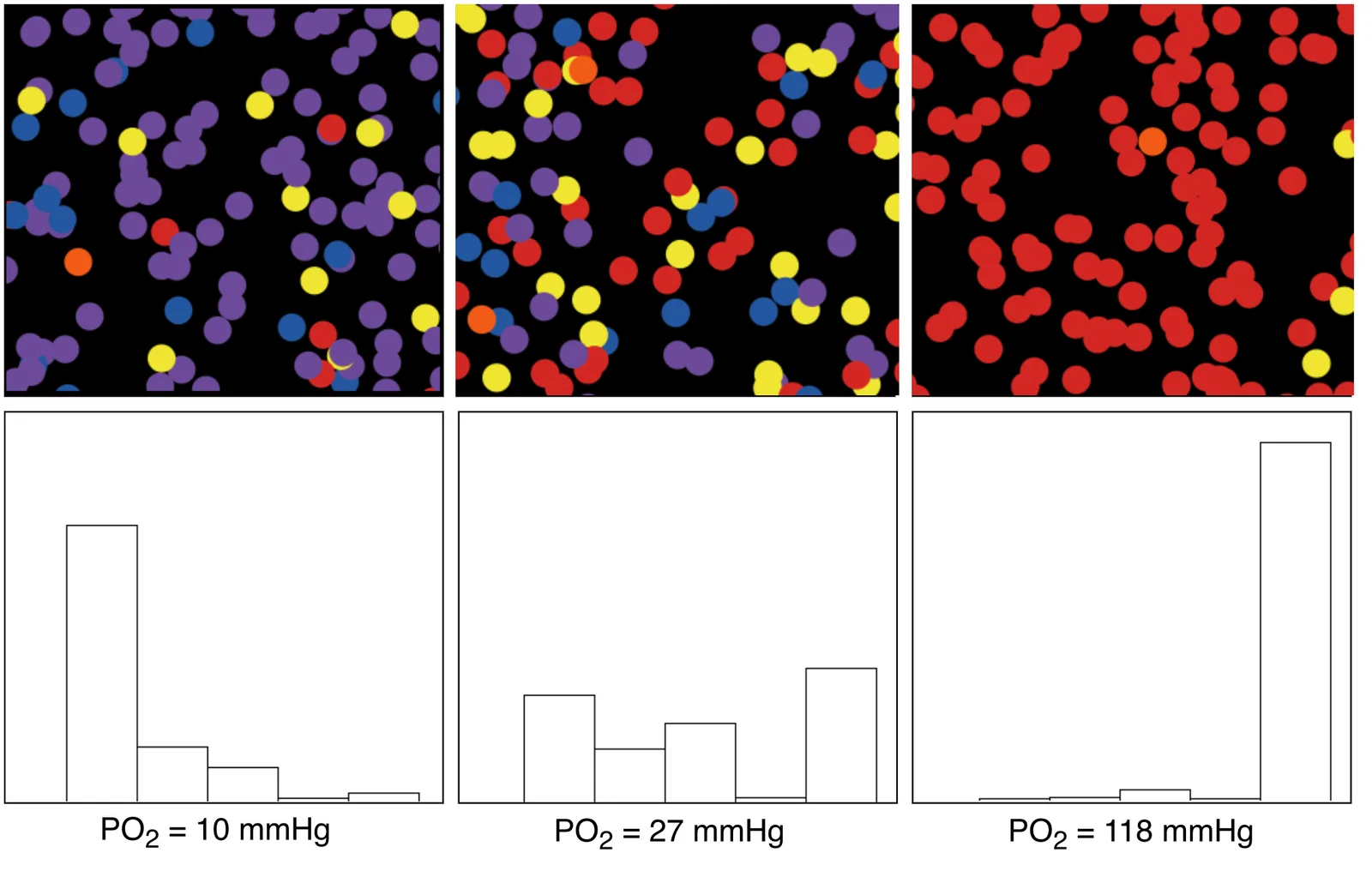
Hb molecules with color-coded states (top row) and corresponding histograms of states (bottom row) from *n* = 0 (left) to *n* = 4 (right) for three different *P*O_2_ settings.

In order to develop confidence in the performance of the ABM, we averaged saturation values generated by the ABM over 1000 time steps for 1000 and 75 Hb molecules respectively at eight *P*O_2_ values ranging from 0 to 140 mmHg in 20 mmHg increments. The mean saturation showed near-identical values between the two Hb numbers but the standard deviation (SD) was substantially larger for the 75 molecules as shown in **Table 2**. This result is particularly important as it demonstrates stochasticity.

**Table 2:** *S*_O2_ presented as mean ± standard deviation (SD) over 1000 time ticks as a function of *P*O_2_, run for N = 1000 and N = 75 Hb molecules respectively.

PO_2_ (mmHg)	N=1000		N=75	
	Mean *S*_O2_ (%)	SD	Mean *S*_O2_ (%)	SD
0	0.0	0.0	0	0
20	30.9	1.2	31	4
40	75.0	1.1	75	4
60	90.0	0.8	90	3
80	94.9	0.5	95	2
100	96.9	0.4	97.0	1.6
120	97.9	0.3	98.0	1.2
140	98.5	0.3	98.5	1.0

Means are almost identical for the two values of N but SD values are substantially larger for N = 75 than for N = 1000.

Given a mean cell hemoglobin (MCH) of about 30 pg per erythrocyte, and using Avogadro’s number (≈ 6.022 × 10^23^ mol^−1^) and the molar mass of Hb (≈ 6.45 × 10^4^ g*/*mol), yields a total number of Hb molecules per red blood cell of about 280 million. With a typical red blood cell concentration of 5 × 10^9^ cells per ml, there are approximately 1.4 × 10^18^ Hb molecules per ml of blood. It is instructive for students to do these calculations themselves.

As the facilitated exploration of the model should clearly leave students with an impression of how large numbers tend to average out stochastic fluctuations, they should easily recognize that in real-world measurements, stochastic effects are far below the detection limit, and any observed rapid fluctuations in *S*_O2_ are due to measurement noise. Students should be able to translate this to other emergent macroscopic properties like temperature, pressure, or radioactive decay, which depend on the stochastic behavior at the microscopic level.

To further verify that the stochastic output of the ABM was producing realistic results, we plotted an oxyhemoglobin equilibrium curve for 1000 Hb molecules from the data in **Table 2** as shown in **Figure 5**, by fitting the Hill model (Hill, 1910) expressed in its modern form as Equation 4, to the data. The fit produced a sigmoidal curve with Hill parameter estimates of *n_H_* = 2.65 ± 0.06 and *P*_50_ = 26.8 ± 0.4 mmHg, which are well within the range determined for human hemoglobin under standard conditions (Clerbaux et al., 1993).

**Figure 5:**
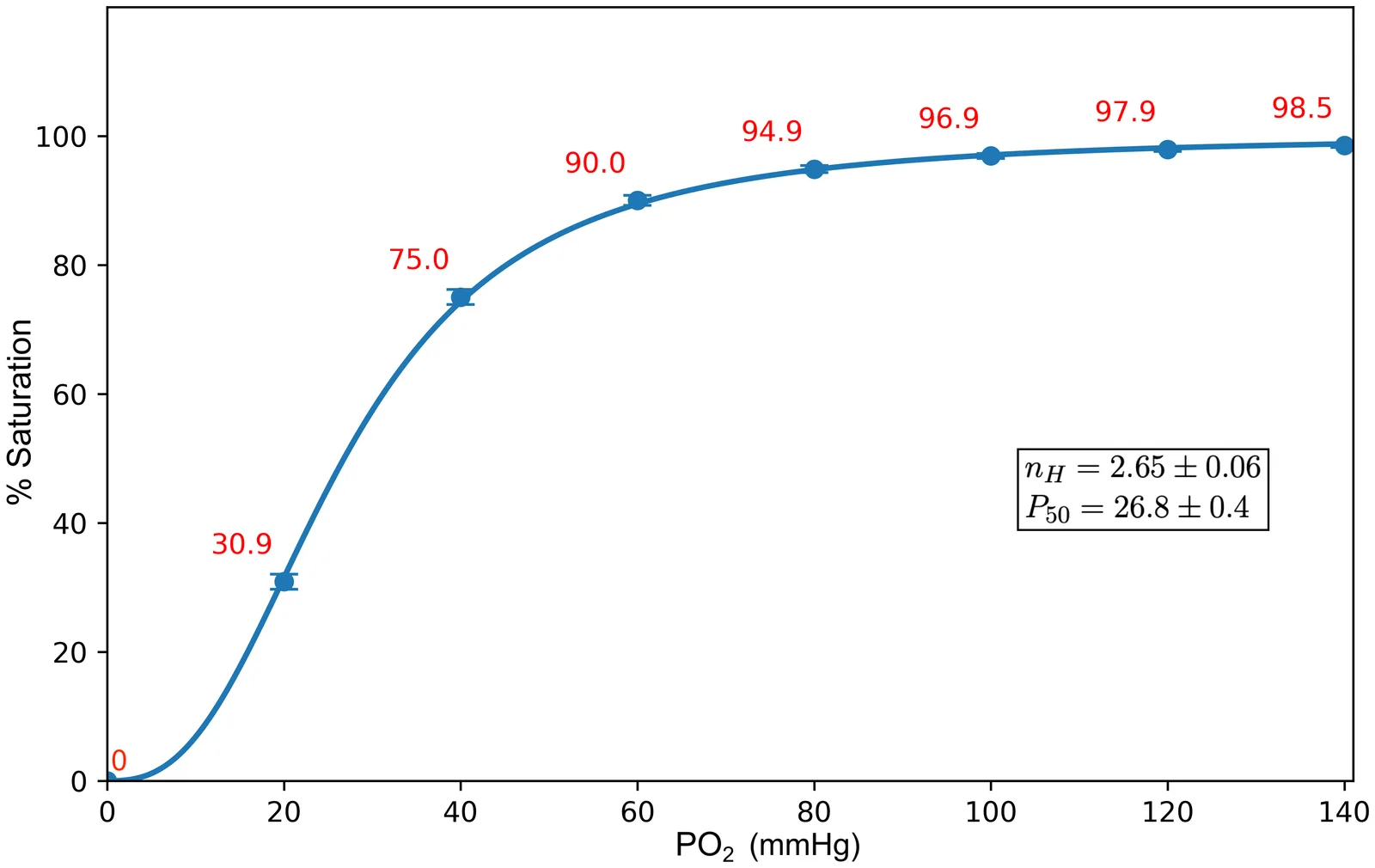
Oxyhemoglobin equilibrium curve fitted to the Hill model from mean saturations (the data points) generated by the ABM outputs at eight *P*O_2_ values measured over 1000 time ticks for N = 1000 Hb molecules. The error bars indicate one standard deviation. Fitted Hill parameters are shown in the legend.

(4) $$S_{O2}=\frac{{\left(PO_{2}\right)}^{n_{H}}}{{\left(P_{50}\right)}^{n_{H}}+{\left(PO_{2}\right)}^{n_{H}}}\times 100$$

### 3.2 Quantitative analysis of quiz performance

The McNemar Test for changes in paired nominal data for the 18 questions *A* → *R* was computed in R (version 4.6.0; R Core Team, 2026) to compare the change between pre-quiz and post-quiz results for the 31 participants. As shown in **Table 3**, the post-quiz averages for all 18 questions showed an improvement over the pre-quiz responses, with 11 of the 18 questions reaching statistical significance at the 5% level or better. A graphical representation of the pre-quiz and post-quiz means is shown in **Figure 6**.

**Table 3:** McNemar test for changes in paired nominal data for the 18 questions *A* → *R*.

Question	Changed		Unchanged		Mean %			
	Regressed	Improved	Correct	Incorrect	Pre-test	Post-test	*p*	Significance
A	0	16	11	4	35.5	87.1	0.00003	***
B	1	15	11	4	38.7	83.9	0.00052	***
C	0	8	22	1	71.0	96.8	0.00781	**
D	1	7	21	2	71.0	90.3	0.07031	
E	1	13	10	7	35.5	74.2	0.00183	**
F	0	17	13	1	41.9	96.8	0.00002	***
G	2	14	10	5	38.7	77.4	0.00418	**
H	2	13	7	9	29.0	64.5	0.00739	**
I	0	7	24	0	77.4	100.0	0.01563	*
J	3	11	8	9	35.5	61.3	0.05737	
K	2	11	13	5	48.4	77.4	0.02246	*
L	1	2	27	1	90.3	93.5	1.00000	
M	2	12	16	1	58.1	90.3	0.01294	*
N	5	7	13	6	58.1	64.5	0.77441	
O	3	11	12	5	48.4	74.2	0.05737	
P	0	5	24	2	77.4	93.5	0.06250	
Q	2	8	20	1	71.0	90.3	0.10938	
R	3	13	12	3	48.4	80.6	0.02127	*

Regressed represents the number of answers that were correct in the pre-quiz but incorrect in the post-quiz. Improved represents the number of answers that were incorrect in the pre-quiz but correct in the post-quiz. Unchanged Correct and Incorrect represent answers that remained correct or incorrect respectively in the pre- and post-quiz. Significance is shown as *p* ≤ 0.05 (∗), *p* ≤ 0.01 (∗∗), and *p* ≤ 0.001 (∗∗∗).

**Figure 6:**
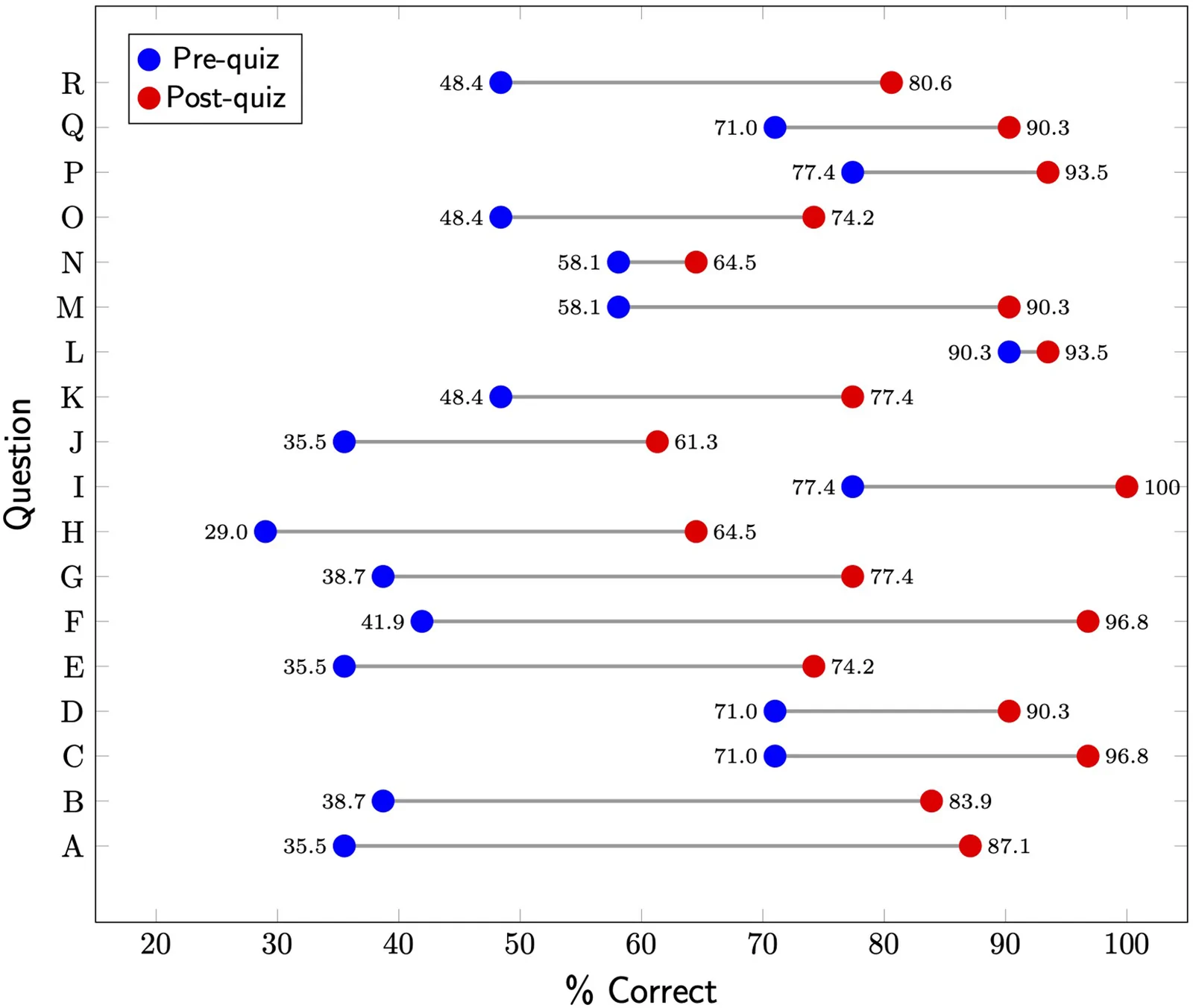
Pre-quiz (blue) and post-quiz (red) mean scores for each of the 18 questions *A* → *R*, averaged over the 31 participants.

A paired samples t-test for n = 31 participants was performed in R (version 4.6.0; R Core Team, 2026) on percentage scores aggregated across the 18 questions. The pre-quiz and post-quiz distributions of percentage scores are summarized in **Figure 7**. The mean scores for the pre-quiz and post-quiz were 54.1% and 83.2% respectively, resulting in a mean difference of 29% (standard deviation = 18.6%, standard error = 3.3% and 95% CI 22.2% to 35.8%). With degrees of freedom (*df*)*, n* − 1 = 30, the t-statistic was 8.7 with a negligible p ≈ 10^−9^. There was a robust effect size with Cohen’s *d_z_* = 1.56.

**Figure 7:**
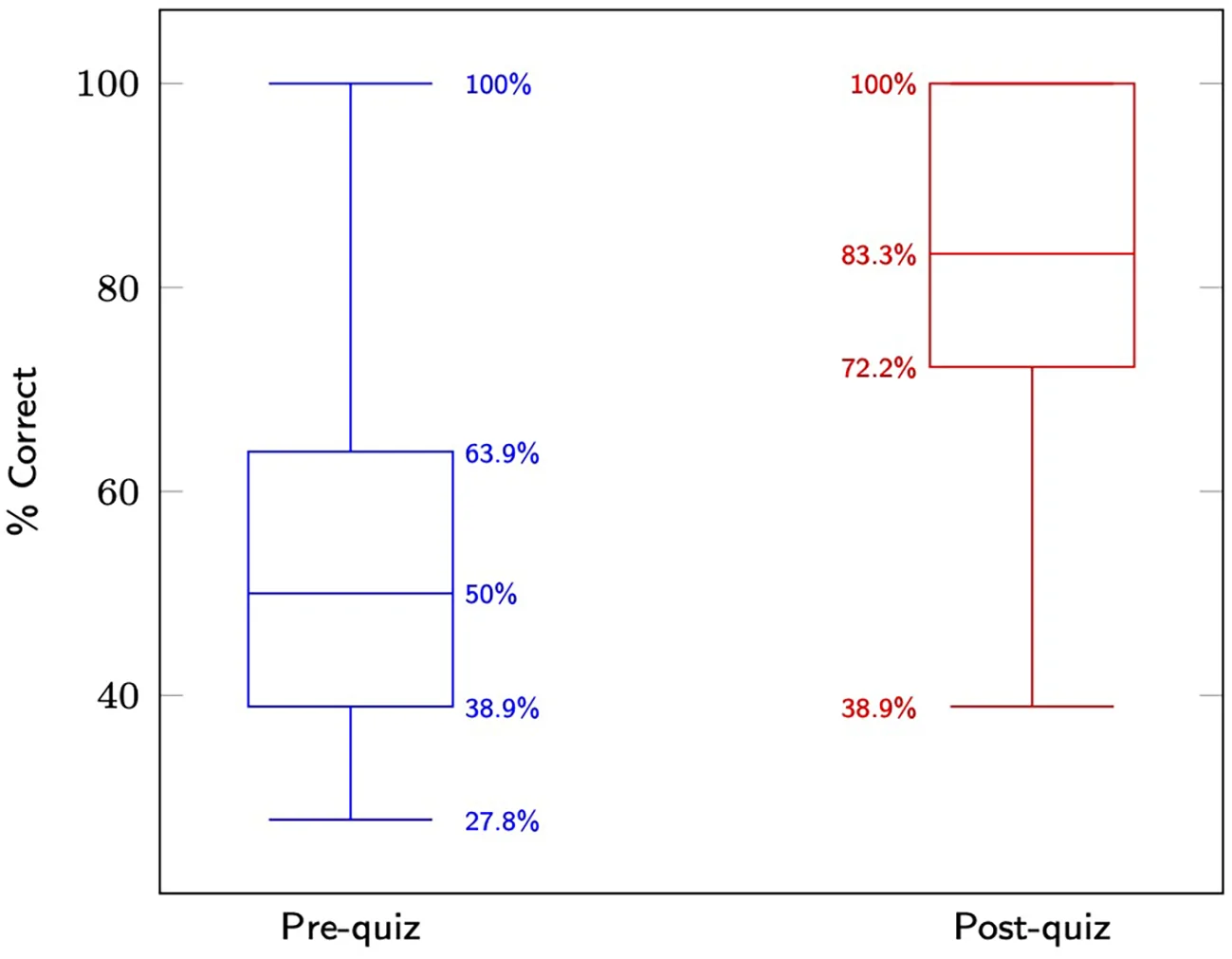
Box-and-whisker plots of the pre-quiz (blue) and post-quiz (red) scores averaged over all 18 questions for the 31 participants. The boxes extend from the first quartile (*Q*_1_) to the third quartile (*Q*_3_), with the median (*Q*_2_) shown as the horizontal line in the box. The interquartile range is defined as IQR = *Q*_3_− *Q*_1_. The whiskers extend to the smallest and largest non-outlying observations. There were no outliers, defined as observations lying outside of the range *Q*_1_− 1.5 × IQR and *Q*_3_ + 1.5 × IQR.

### 3.3 Qualitative analysis of focus groups

Three inductive themes were identified from the focus group transcripts. These themes described how participants experienced the modeling activity, how they constructed understanding through interaction with the model, and how the activity contributed to changes in their conceptual understanding.

#### 3.3.1 Theme 1: knowledge construction through active exploration

Participants consistently described the interactive nature of the agent-based model as central to their learning experience. Rather than experiencing the model as a passive representation of physiological processes, participants described actively constructing understanding through manipulation, experimentation and observation. Two related subthemes were identified: learning through active exploration of the model, and revising prior mental models through interaction.

##### 3.3.1.1 Subtheme 1.1: learning through active engagement and experimentation

Participants repeatedly emphasized the value of being able to manipulate physiological variables, explore different scenarios and observe the consequences of their changes. The opportunity to interact directly with the model appeared to allow participants to develop understanding through experimentation:

“…the model is really cool because you get to change it and adjust it to those like abnormal values and see the actual impact of it.” [A1]

“I feel like it’s simpler for us to learn through interaction.” [B2]

Participants contrasted this exploratory approach with more traditional forms of learning, suggesting that direct interaction enabled forms of understanding that were difficult to achieve through more passive learning.

“I think that anything that’s interactive and that you can sort of change to see results and is much more useful than reading and watching a lecture and even looking at examples…” [B3]

The exploratory nature of the activity was also described as a particularly valuable feature of the learning experience.

“So I really loved the like explorative aspects of it.” [D4]

##### 3.3.1.2 Subtheme 1.2: revising prior mental models through interaction

Beyond supporting exploration, interaction with the model prompted participants to reconsider and revise their previous understanding of oxygen–hemoglobin binding. Several participants described recognizing that their prior mental models were incomplete or incorrect after observing the behavior of the simulated system, as one participant explained:

“I I knew I didn’t know the full extent of oxyhemoglobin binding, but I thought I kind of knew what was going on. But once we got to interact with the model, I realized that my previous understanding was completely incorrect.” [D5]

Participants also described moving away from static representations toward a more mechanistic understanding based on observing the consequences of changing physiological parameters:

“Yeah, I feel like we shifted away from just looking at textbook graphs and pictures, to actually adjusting values ourselves, like the partial pressure of oxygen that … that really helped my understanding.” [D6]

Together, these accounts suggest that the model functioned not simply as a visual aid, but as a tool for active knowledge construction. By allowing participants to manipulate variables and observe emergent behavior, the activity supported the development and refinement of conceptual models of oxygen–hemoglobin binding.

#### 3.3.2 Theme 2: affective responses associated with intellectual engagement

Participants frequently described the learning experience as involving both emotional and intellectual engagement. Their accounts suggested that conceptual change was not simply a process of acquiring new information, but was accompanied by curiosity, excitement, moments of insight and reflection on previously held assumptions. Four related subthemes were identified: epistemic pleasure, recognition of previously hidden misconceptions, a desire to communicate new understanding, and enthusiasm for transformative and integrative insights.

##### 3.3.2.1 Subtheme 2.1: epistemic pleasure and moments of discovery

Participants frequently described the modeling activity as fascinating, exciting and intellectually rewarding. These responses reflected enjoyment of the process of understanding itself, rather than simply satisfaction from achieving a correct answer:

“I just thought it was the coolest thing.” [A1]

For some participants the learning experience was fascinating and exciting:

“Yeah, for me it was just quite fascinating. I, I didn’t feel uncomfortable at any … at any time. I just genuinely just felt fascinated. It was less uncomfortable and more exciting.” [C1]

“For me it was kind of exciting.” [C4]

Several participants described moments of sudden conceptual clarity during the activity, often referring to these experiences as “light bulb” or “Eureka” moments. These moments appeared to occur when participants were able to connect their previous uncertainty with the behavior observed in the model.

“I actually had many light bulb moments because in the pre-quiz I wrote a lot of things hey. I wrote a lot of the things that were … that I wasn’t sure about. But then once I saw everything playing out, even before you guys explained it, I was thinking, oh OK, that was wrong, that was right. Like I could relate the questions that were asked before with almost zero knowledge and then I could relate it to what was going on. I could see where I went wrong, see where I went right…” [C6]

For some participants, this intellectual engagement extended beyond the facilitated session and motivated further independent exploration:

“But I was interested and I went to look … look it up afterwards and kind of understood it a bit further.”[C6]

Participants also described developing a new appreciation for the complexity and interest of oxygen– hemoglobin binding.

“I didn’t realize how interesting oxyhemoglobin binding would be, so I found that quite cool.” [D3]

##### 3.3.2.2 Subtheme 2.2: recognizing and confronting previous misconceptions

Participants frequently reflected on recognizing limitations in their prior understanding. A recurring feature of their accounts was the realization that they had previously been confident in explanations that were incomplete or incorrect. The activity appeared to make previously hidden misconceptions visible, allowing participants to critically examine their own understanding:

“The thing is, before I didn’t even know what I didn’t know.” [A1]

One participant described the experience as prompting broader reflection on the reliability of their own confidence in other areas of knowledge.

“So before I went and I thought that I understood it, like I thought I was 100% sure. And then in that moment when he was explaining, I realized that I didn’t know anything. I didn’t know anything. And then I understood it. But when I went back, I was kind of disappointed in myself. I thought that maybe there are also other things that I’m confident in and I don’t really, really know.” [B1]

Similarly, other participants explicitly described recognizing that their previous understanding had been based on incorrect assumptions.

One participant commented that the session caused them to re-evaluate their initial level of confidence:

“I think I was falsely confident before the session and I thought I understood it.” [B6]

Another participant explained that it challenged their preconceived ideas:

“…for me it got me to think really that OK my preconceived ideas were incorrect.” [C4]

##### 3.3.2.3 Subtheme 2.3: desire to communicate new understanding

Several participants described sharing their newly developed understanding with others following the session. This desire to communicate their insights suggests that the learning experience generated personal significance beyond the immediate educational context.

“I actually explained to my mom what I was doing and she doesn’t work in healthcare.” [A2]

“…I think it’s easy to like get a few fixed perspective[s] of how it works, but I think after the session I was able to understand and I was even telling my friends…” [D4]

Participants also expressed enthusiasm for encouraging future students to engage with the activity.

“…if you’d ask like one of us, if we’d be willing like to go and explain like how much it benefited and how much we actually understood, that could help bring people to use it.” [B6]

Together, these accounts indicate that the modeling activity generated more than cognitive gains alone. Participants described a learning experience characterized by curiosity, intellectual enjoyment, critical reflection and a motivation to share newly constructed understanding with others.

##### 3.3.2.4 Subtheme 2.4: enthusiasm for transformative and integrative insights

Several participants described a marked difference between their understanding before and after the session. Participants also described gaining a more coherent understanding of oxygen binding and the oxyhemoglobin equilibrium curve.

“…I felt personally…. that I gained a much greater understanding of how oxygen binds hemoglobin and how it disassociates. And I understood the curve itself a lot better.” [C1]

The ability to manipulate physiological variables and observe emergent behavior appeared to contribute to these transformative experiences.

“You know, during the session I had a lot of, uh, like wow moments where I was like, oh, I can adjust this like this, and also especially when we moved the partial pressure to zero and I saw all the hemoglobin going one color … that was really helpful with my understanding.” [D6]

Together, these accounts suggest that the intervention facilitated a conceptual transition from simplified representations of oxygen transport toward a more mechanistic understanding of oxygen–hemoglobin interactions.

Some participants described being able to apply their new understanding to clinical or physiological scenarios.

“…it got to a point where now if somebody were to ask me about any sort of like hemoglobin related disorders, I could begin to simulate what would happen in my mind.” [C5]

Others described connecting the concept to wider physiological systems.

“I think because we’re doing endocrine as well in physiology … it kind of gives you an idea of how everything’s connected.” [D1]

Participants also recognized links between molecular behavior, physiology and broader scientific principles.

“I think it sort of like, um, puts it into the whole world of, I guess physics, where we’ve got molecules that are bouncing off each other.” [D4]

These accounts indicate that participants were not only developing a more accurate understanding of oxygen–hemoglobin binding, but were also beginning to integrate this understanding within wider conceptual frameworks.

#### 3.3.3 Theme 3: development of mechanistic understanding of oxygen–hemoglobin binding

In addition to the inductive themes identified from participants’ accounts, the transcripts were examined deductively for evidence of key scientific concepts that the intervention was designed to address. Four interconnected concepts were identified: stochasticity, statistical averaging of molecular states, dynamic equilibrium, and a shift away from the deterministic “goods train” model of oxygen transport. Together, these concepts represent a shift toward a mechanistic understanding of oxygen–hemoglobin binding as an emergent process arising from the behavior of individual hemoglobin molecules.

##### 3.3.3.1 Subtheme 3.1: stochasticity of oxygen binding

Participants described developing an understanding that oxygen binding and release are probabilistic processes rather than predictable, deterministic events. Several participants contrasted their previous assumptions with the dynamic behavior observed in the model, where individual hemoglobin molecules could bind and release oxygen continuously.

One participant described this as a Eureka moment:

“But I think I had like as you said the Eureka moment when it was when you saw like the little molecules and how one went from purple to yellow to red, I think that was very like, OK, so that’s how that works.” [A2]

Participants specifically identified the random nature of oxygen binding and release as a new conceptual insight.

“…the whole concept of it dissociating randomly and then picking it back up again, that was also something that I was introduced to for the first time.” [C5]

The model also challenged the assumption that all hemoglobin molecules respond identically under a given physiological condition.

“I just feel like another misunderstanding that I had before was if the hemoglobin was in the area that like required a lot of oxygen, like all the hemoglobin would release its oxygen … I feel like that’s like when I saw it and how the colors were constantly changing.” [C7]

Participants described recognizing that oxygen binding is therefore an ongoing and changing process rather than a sequence of fixed states.

“It’s different. It’s dynamic. It’s always changing. I feel like, you know, that’s what I took out and that is what I would say to someone.” [D2]

##### 3.3.3.2 Subtheme 3.2: statistical averaging of molecular states

Participants also described recognizing that measurable physiological quantities, such as oxygen saturation, represent averages across populations of hemoglobin molecules rather than the state of an individual molecule. This represented an important shift from interpreting macroscopic measurements as direct representations of individual molecular behavior.

“But then we also learned that you’re kind of looking at all of the molecules that exist in one go … That’s how you get the 85, 86% saturation, the weighted averages of everything, which is quite interesting for me.” [A6]

Another participant described this realization as a particularly important conceptual breakthrough.

“…I saw the model and I saw like that but it’s an average that like the number you get like when you’re testing in the hospital, it’s like an average for the hemoglobin molecules. I think that was like the biggest click for me.” [D1]

These accounts suggest that the model helped participants connect observable physiological measurements with the underlying distribution of molecular states that produces them.

##### 3.3.3.3 Subtheme 3.3: dynamic equilibrium

Participants described developing an understanding of oxygen binding as a dynamic equilibrium involving simultaneous association and dissociation, rather than as a static process in which hemoglobin simply switches between oxygen-bound and oxygen-free states.

“…oxygen doesn’t like just let go essentially of the hemoglobin, it like also binds to it like through that dynamic equilibrium.” [A2]

For some participants, the model provided a framework that made a previously abstract concept understandable.

“It makes sense in the context of equilibrium now, but before it was just magic.” [B3]

Participants also described recognizing that multiple molecular states coexist simultaneously rather than occurring as discrete sequential stages.

“So like you have a … can provide a different understanding that it is a dynamic equilibrium not a constant thing like it’s either like 100% or it’s 0% or 25% rather they exist in those states at the same time.” [B4]

This understanding was linked directly to observation of the molecular behavior represented in the model.

“…so you can actually see that it is in fact a dynamic equilibrium regardless of how many hemoglobin[s] that you see or that you have.” [B5]

##### 3.3.3.4 Subtheme 3.4: moving beyond the “goods train” model

A recurring feature of participants’ accounts was the recognition that their previous understanding of oxygen transport had been based on a simplified, deterministic model in which hemoglobin acted like a vehicle that collected oxygen in the lungs and delivered it to tissues. The intervention appeared to challenge this “goods train” misconception by demonstrating the probabilistic and dynamic nature of oxygen binding.

“…I also had the idea or I had the notion that at the concentration at the lungs that’s fully saturated and then when it gets to the tissue it offloads everything.” [B2]

Participants described previously interpreting the oxygen–hemoglobin equilibrium curve as representing fixed points at which oxygen was either picked up or released.

“I was thinking before that the curve was sort of – at this oxygen concentration hemoglobin picks up, at this concentration hemoglobin puts it down.” [B3]

The model helped participants replace this deterministic interpretation with an understanding based on continuous molecular interactions.

“So the introduction to the more probabilistic nature of oxygen binding … I was under the impression that hemoglobin goes to the lungs. It picks up 4 oxygen molecules, holds them tight. It goes to the tissues, releases them.” [C5]

Together, these accounts demonstrate that participants developed a more mechanistic understanding of oxygen–hemoglobin binding. Rather than viewing oxygen transport as a deterministic process, participants described recognizing that macroscopic physiological behavior emerges from dynamic, stochastic interactions occurring at the molecular level.

### 3.4 Integrating the findings

The quantitative and qualitative findings demonstrated a high degree of convergence, with the qualitative data providing insight into the nature of the conceptual changes underlying the observed improvements in assessment performance. While the quantitative analysis demonstrated significant gains in participants’ responses following the intervention, the qualitative findings helped to explain how these changes occurred by revealing shifts in participants’ mental models, their engagement with the learning process, and their developing understanding of the molecular mechanisms underlying oxygen–hemoglobin binding.

Integration of the quantitative and qualitative findings is presented in **Table 4**. The combined findings indicate that the agent-based modeling activity supported more than an increase in factual knowledge. Rather, participants described a transition toward a mechanistic understanding of oxygen–hemoglobin binding, characterized by recognition of stochasticity, dynamic equilibrium and statistical averaging of molecular micro-states. The convergence between improved quantitative performance and qualitative evidence of active knowledge construction, epistemic engagement and transformative learning provides complementary evidence for the educational value of this intervention.

**Table 4:** Integration of quantitative and qualitative findings demonstrating changes in participants’ conceptual understanding following the agent-based modeling intervention.

Quantitative finding	Qualitative evidence	Integrated interpretation
Improvement across all 18 quiz questions, with 11 showing statistical significance on the McNemar test.	Participants described developing new understanding through interaction with the model, recognizing previous misconceptions, and experiencing transformative and integrative changes in their understanding.	The quantitative improvement reflected conceptual change rather than simple factual recall. The pattern of improvement and reported changes in understanding suggests that stochastic oxygen–hemoglobin binding may represent a threshold concept in physiology education.
Mean scores increased from 54.1% to 83.2% with *t*(30) = 8.7 (*p* = 10^−9^), and effect size *d_z_*= 1.56.	Participants described substantial shifts in their understanding of oxygen–hemoglobin binding.	The magnitude of improvement was consistent with transformative learning experiences and substantial changes in participants’ conceptual frameworks.
Improved understanding of oxygen binding concepts as evidenced by the large positive changes in mean scores for every question, addressing each of the four aspects of the process.	Participants described stochastic binding, dynamic equilibrium, and statistical averaging of molecular states, as well as a shift away from the “goods train” model.	The intervention supported development of a mechanistic understanding of oxygen–hemoglobin binding and a transition away from deterministic explanations toward a more dynamic and probabilistic understanding.
Almost all (28 of 31) participants improved on the post-quiz. The remaining three all had high pre-quiz scores, one with a perfect score – two remained the same and one showed a small decrease.	Participants valued manipulating variables, exploring scenarios, and observing consequences.	The model supported active construction of knowledge through experimentation and exploration.

## 4 Discussion

The quantitative and qualitative findings converged to demonstrate that facilitated exploration of the agent based model promoted a substantial improvement in participants’ understanding of oxygen–hemoglobin binding. Quantitatively, participants showed significant gains across most questions, with a large overall effect size (*d_z_*= 1.56). Qualitatively, participants consistently attributed these gains to actively manipulating the model, observing the consequences of changing physiological variables, and discussing their observations during the facilitated session. Participants used terms such as “light bulb” or “Eureka” moments to described their experience of sudden insights as they came to appreciate concepts such as stochasticity, dynamic equilibrium, and statistical averages across a population of hemoglobin molecules. In this process, many abandoned the deterministic “goods train” misconception of oxygen transport.

Beyond improved performance, the intervention elicited strong positive affective responses. Participants described fascination, curiosity, excitement and enjoyment, frequently expressing a desire to share their new understanding with others. Such responses are consistent with active learning (Borrego et al., 2018; Jaber and Hammer, 2016). Epistemic curiosity has been described as the pleasurable drive to acquire knowledge (Litman, 2005), while Pekrun et al. (2002) characterizes this emotional state as epistemic enjoyment. We suggest that the broader term *epistemic pleasure*, consistent with Cohen (2020), better captures the range of affective responses observed in this study.

These findings are consistent with theories of active learning, which emphasize learners’ active construction of understanding rather than the passive reception of information (Petress, 2008). Active learning has repeatedly been associated with improved educational outcomes (Freeman et al., 2014; Michael, 2006). More specifically, the intervention aligns with Papert’s constructionism, in which participants construct knowledge through interaction with meaningful computational artefacts (Harel and Papert, 1991). NetLogo was explicitly developed to support this style of learning (Wilensky, 1999a; Blikstein et al., 2005), and we have previously argued that computational modeling in medical education may constitute a constructionist activity (Rubin et al., 2026). Nevertheless, the present study was not designed to isolate the contributions in active learning per se, as will be elaborated upon under the discussion on limitations.

Participants also demonstrated increased metacognitive awareness by recognizing limitations in their previous understanding despite initial confidence. This reflective self-criticism suggests that the facilitated modeling activity not only replaced misconceptions with more accurate explanations, but also encourages participants to evaluate the adequacy of their own mental models.

The findings further suggest that stochastic oxygen–hemoglobin binding may represent a threshold concept. Dynamic equilibrium is likely to fall into the category of troublesome knowledge as defined by Perkins (2006), and ABM is well suited to exploration (Stieff and Wilensky, 2003; Andriani et al., 2021). Consistent with the framework of Meyer and Land (2003, 2005), the strongest qualitative evidence related to transformative learning, with participants describing substantial conceptual shifts in their understanding.

Evidence for irreversibility, boundedness and liminality was less apparent, largely because the focus groups were conducted only eight days after the intervention. Longitudinal studies are therefore needed to determine whether these additional characteristics emerge over time.

This study has several limitations. The cohort was small, self-selected and drawn from a single institution, limiting generalizability, although the before–after design allowed participants to serve as their own controls.

As this study seeks to investigate the effectiveness of a facilitated exploration of the agent-based model in the context of understanding the behavior of oxyhemoglobin interactions at the microstate level, it is not possible to draw conclusions as to the relative merits of various instructional approaches – this may be considered in future studies.

An additional limitation of this study is that the facilitated ABM session comprised several interacting educational components, and the study design does not allow their individual contributions to be distinguished. Participants actively explored and manipulated the ABM while simultaneously engaging in facilitator-led questioning, discussion, and explanation. The observed improvement in post-intervention responses may therefore have resulted from the ABM itself, from the active engagement associated with exploring the model, from the facilitator’s explanations and discussion, or from the interaction of these components. Similarly, the qualitative responses of students included comments on both active engagement and the facilitation, however this is insufficient to distinguish between their relative contributions.

Consequently, the present findings should not be interpreted as demonstrating that in active learning, the ABM, or facilitator-led discussion individually produced the observed improvement. Rather, our findings demonstrate an association between participation in the facilitated exploration of the ABM as a combined educational intervention, and improved performance on the post-intervention assessment. This distinction is particularly important given the broader literature supporting active learning, because the present study did not include a comparator that would allow the specific contributions in active learning to be isolated. Similarly, the study does not establish whether the facilitated ABM is more effective than a conventional or appropriately designed lecture addressing the same conceptual difficulties.

Disentangling these contributions would require a different experimental design. Future studies could compare facilitated ABM exploration with an appropriately matched instructional intervention, and potentially examine ABM exploration with and without facilitation. Such designs could determine whether the model provides an additional educational benefit beyond explanation alone and whether facilitation enhances the value of model-based exploration. These questions are important but fall beyond the scope of the present exploratory investigation.

Another important limitation is that the study was not designed to investigate differences in participants’ responses to the intervention. Although substantial improvement occurred overall, some students continued to answer particular questions incorrectly. As individual-level educational and learning-preference data were not collected, we cannot determine whether differences in prior knowledge, experience, engagement, or learning approaches contributed to these responses. Residual incorrect responses therefore do not necessarily indicate that the intervention was ineffective for particular students, but rather that conceptual change was incomplete. Future studies should investigate whether participant characteristics are associated with benefit from facilitated ABM-based learning.

Regarding the design of the ABM, in this study, we were restricted to using the coefficients of Chou and Lee (2023) which were specific to only one oxyhemoglobin equilibrium curve. Modeling left and right shifts of the curve in response to CO_2_, temperature, pH and 2,3–DPG is likely to greatly enhance the utility of this approach and it is left for future work.

Following the positive responses from participants, a demonstration to laboratory coordinators and senior faculty from the Department of Physiology at our institution resulted in a decision to integrate this activity into the respiration laboratory for the current semester. Further studies on this cohort may be considered in the future, which could address some of the limitations of this study such as self-selection bias.

## 5 Conclusion

Facilitated exploration of the agent-based model produced substantial improvements in conceptual understanding while promoting active knowledge construction, epistemic pleasure and reflective metacognition. The convergence of quantitative learning gains and qualitative evidence of conceptual transformation suggests that agent-based modeling is a promising approach for teaching the stochastic nature of oxygen–hemoglobin binding and other complex physiological processes that are difficult to represent using traditional approaches. Further research should address the limitations of the present study, including the durability and transferability of these conceptual changes.

The broader potential for the use of ABM in physiology education and research should be explored. Numerous macroscopically observable processes are governed by microstates that display stochastic properties and are therefore amenable to ABM. These include the ubiquitous properties of molecular diffusion and membrane transfer, hormone and drug binding to receptors, and cell motility and dynamics in growth, cancer, immune response, coagulation, and wound healing. The oxygen–hemoglobin model illustrates how such molecular-level microstates can generate emergent physiological behavior, and this common modeling framework may be transferable across these diverse physiological systems.

## Data Availability

The original contributions presented in the study are included in the article/**Supplementary Material**. Further inquiries can be directed to the corresponding author.

## Supplementary material

A video showing the operation of the ABM can be found at: 10.6084/m9.figshare.33282099
