# Supplementary material for: Stochastic dynamics of oxygen-hemoglobin binding: insight through facilitated exploration of an agent-based model
Source: Front Physiol. 2026 Oct 1;17:1953414. doi: 10.3389/fphys.2026.1953414 (PMC13714974; doi:10.3389/fphys.2026.1953414)
Supplement: Supplementary file 1 [file DataSheet1.pdf]

Pre-quiz

Your anonymous number

Please indicate TRUE or FALSE after each statement.

A) At the lungs where the partial pressure of oxygen is high, oxygen molecules only bind to haemoglobin molecules but do not dissociate from haemoglobin molecules.

B) At the peripheral tissues where the partial pressure of oxygen is low, oxygen molecules only dissociate from haemoglobin molecules but do not bind to haemoglobin molecules.

C) An individual haemoglobin molecule can only be 0, 25%, 50%, 75% or 100% saturated with O_2.

D) In reality, the O_2-haemoglobin dissociation curve can only have saturations of 0, 25%, 50%, 75% or 100% We just join the points to make it easier to read.

E) If a severely hypoxic person has an oxygen-haemoglobin saturation of 75%, then each molecule of haemolobin has exactly 3 molecules of oxygen bound to it.

F) For blood with dissolved oxygen at a high partial pressure of 153 mmHg, there will be some haemoglobin molecules with no oxygen bound to them, while other haemoglobin molecules will have 1, 2, 3 or 4 oxygen molecules bound to them.

G) When a haemoglobin molecule with oxygen bound to it reaches the peripheral tissues, it releases all of its oxygen molecules.

H) If a specific haemoglobin molecule had three oxygen molecules bound to as it leaves the high partial pressure of oxygen milieu of the lungs, then it could not have 4 oxygen molecules bound to it in the low partial pressure milieu of the peripheral tissues.

I) Every haemoglobin molecule in a sample at fixed partial pressure of oxygen will have the same number of oxygen molecules bound to it.

J) If a specific haemoglobin molecule has 2 oxygen molecules bound when it is in a milieu where the saturation (SpO_2) is 85%, then if the SpO_2 changes to 98% it would always have more than 2 oxygen molecules bound to it.

K) If the saturation (SpO_2) is 93% and due to a pH change, the O_2-haemoglobin dissociation curve shifts to the left but with the same oxygen partial pressure, then every individual molecule of haemoglobin will have more oxygen molecules bound to it than it did before the shift.

L) The percentage oxygen saturation (SpO_2) for a patient in ICU with respiratory failure is 83%. This percentage is a weighted average of the individual saturation levels of the individual percentage binding of all the haemoglobin molecules.

M) A haemoglobin molecule exposed to a fixed partial pressure of oxygen, will have a fixed number of oxygen molecules bound to it, and moreover, it will be the same oxygen molecules.

N) At a fixed partial pressure, the average oxygen saturation (SpO_2) for a very small number of haemoglobin molecules (e.g. a few hundred), is smaller than for a very large number (e.g. many billions).

O) At a fixed partial pressure, the oxygen saturation is much more variable for a very small number of haemoglobin molecules (e.g. a few hundred), than for a very large number (e.g. many billions).

P) At a given oxygen partial pressure, O_2 molecules continuously bind and dissociate from haemoglobin molecules but the average rate of binding and dissociation is the same.

Q) The average rate of dissolved oxygen binding to haemoglobin and the average rate at which oxygen dissociates from haemoglobin to re-enter solution will become equal (dynamic equilibrium) if the partial pressure of oxygen is held constant.

R) The average rate at which dissolved oxygen binds to haemoglobin and the average rate at which oxygen dissociates from haemoglobin to re-enter solution, will not become equal (dynamic equilibrium) in a right-shifted O_2-haemoglobin dissociation curve, even if the oxygen partial pressure is held constant.

Post-quiz

Your anonymous number

Please indicate TRUE or FALSE after each statement.

A) At the lungs where the partial pressure of oxygen is high, oxygen molecules only bind to haemoglobin molecules but do not dissociate from haemoglobin molecules.

B) At the peripheral tissues where the partial pressure of oxygen is low, oxygen molecules only dissociate from haemoglobin molecules but do not bind to haemoglobin molecules.

C) An individual haemoglobin molecule can only be 0, 25%, 50%, 75% or 100% saturated with O_2.

D) In reality, the O_2-haemoglobin dissociation curve can only have saturations of 0, 25%, 50%, 75% or 100% We just join the points to make it easier to read.

E) If a severely hypoxic person has an oxygen-haemoglobin saturation of 75%, then each molecule of haemolobin has exactly 3 molecules of oxygen bound to it.

F) For blood with dissolved oxygen at a high partial pressure of 153 mmHg, there will be some haemoglobin molecules with no oxygen bound to them, while other haemoglobin molecules will have 1, 2, 3 or 4 oxygen molecules bound to them.

G) When a haemoglobin molecule with oxygen bound to it reaches the peripheral tissues, it releases all of its oxygen molecules.

H) If a specific haemoglobin molecule had three oxygen molecules bound to as it leaves the high partial pressure of oxygen milieu of the lungs, then it could not have 4 oxygen molecules bound to it in the low partial pressure milieu of the peripheral tissues.

I) Every haemoglobin molecule in a sample at fixed partial pressure of oxygen will have the same number of oxygen molecules bound to it.

J) If a specific haemoglobin molecule has 2 oxygen molecules bound when it is in a milieu where the saturation (SpO_2) is 85%, then if the SpO_2 changes to 98% it would always have more than 2 oxygen molecules bound to it.

K) If the saturation (SpO_2) is 93% and due to a pH change, the O_2-haemoglobin dissociation curve shifts to the left but with the same oxygen partial pressure, then every individual molecule of haemoglobin will have more oxygen molecules bound to it than it did before the shift.

L) The percentage oxygen saturation (SpO_2) for a patient in ICU with respiratory failure is 83%. This percentage is a weighted average of the individual saturation levels of the individual percentage binding of all the haemoglobin molecules.

M) A haemoglobin molecule exposed to a fixed partial pressure of oxygen, will have a fixed number of oxygen molecules bound to it, and moreover, it will be the same oxygen molecules.

N) At a fixed partial pressure, the average oxygen saturation (SpO_2) for a very small number of haemoglobin molecules (e.g. a few hundred), is smaller than for a very large number (e.g. many billions).

O) At a fixed partial pressure, the oxygen saturation is much more variable for a very small number of haemoglobin molecules (e.g. a few hundred), than for a very large number (e.g. many billions).

P) At a given oxygen partial pressure, O_2 molecules continuously bind and dissociate from haemoglobin molecules but the average rate of binding and dissociation is the same.

Q) The average rate of dissolved oxygen binding to haemoglobin and the average rate at which oxygen dissociates from haemoglobin to re-enter solution will become equal (dynamic equilibrium) if the partial pressure of oxygen is held constant.

R) The average rate at which dissolved oxygen binds to haemoglobin and the average rate at which oxygen dissociates from haemoglobin to re-enter solution, will not become equal (dynamic equilibrium) in a right-shifted O_2-haemoglobin dissociation curve, even if the oxygen partial pressure is held constant.
